# Loss of Y Chromosome in Peripheral Blood of Colorectal and Prostate Cancer Patients

**DOI:** 10.1371/journal.pone.0146264

**Published:** 2016-01-08

**Authors:** Predrag Noveski, Svetlana Madjunkova, Emilija Sukarova Stefanovska, Nadica Matevska Geshkovska, Maja Kuzmanovska, Aleksandar Dimovski, Dijana Plaseska-Karanfilska

**Affiliations:** 1 Research Center for Genetic Engineering and Biotechnology “Georgi D. Efremov”, Macedonian Academy of Sciences and Arts, Skopje, Republic of Macedonia; 2 Center for Biomolecular Pharmaceutical Analysis, Faculty of Pharmacy, University Ss Cyril and Methodius, Skopje, Republic of Macedonia; University of Cagliari, ITALY

## Abstract

**Background:**

Although age-related loss of chromosome Y (LOY) in normal hematopoietic cells is a well-known phenomenon, the phenotypic consequences of LOY have been elusive. However, LOY has been found in association with smoking, shorter survival and higher risk of cancer. It was suggested that LOY in blood cells could become a predictive biomarker of male carcinogenesis.

**Aims, Methods & Findings:**

To investigate the association of LOY in blood cells with the risk for development of colorectal (CC) and prostate cancers (PC), we have analyzed DNA samples from peripheral blood of 101 CC male patients (mean age 60.5±11.9 yrs), 70 PC patients (mean age 68.8±8.0 yrs) and 93 healthy control males (mean age 65.8±16.6 yrs). The methodology included co-amplification of homologous sequences on chromosome Y and other chromosomes using multiplex quantitative fluorescent (QF) PCR followed by automatic detection and analysis on ABI 3500 Genetic Analyzer. The mean Y/X ratio was significantly lower in the whole group of cancer patients (0.907±0.12; p = 1.17x10^-9^) in comparison to the controls (1.015±0.15), as well as in CC (0.884±0.15; p = 3.76x10^-9^) and PC patients (0.941±0.06; p = 0.00012), when analyzed separately. Multivariate logistic regression analysis adjusting for LOY and age showed that LOY is a more significant predictor of cancer presence than age, and that age probably does not contribute to the increased number of subjects with detectable LOY in cancer patients cohort.

**Conclusion:**

In conclusion, our results support the recent findings of association of LOY in blood cells with carcinogenesis in males.

## Introduction

Loss of Y chromosome (LOY) is a well-known phenomenon that is associated with aging and observed with varying frequencies in bone marrow cells [1,2] or in peripheral blood cells [3,4] from healthy older males. The association of LOY with hematological cancers has been elusive. However, LOY has been reported in leukemias [5,6,7,8] and in patients predicted to have poor response to cancer therapy [9]. Other studies have found the association only in patients who showed LOY in more than 75% [10] or 100% of the affected cells [11]. Recent study investigating LOY in two separate peripheral blood cell fractions found significantly higher frequency of LOY in CD34+ cells in patients with myelodysplastic syndromes compared with elderly men without hematologic diseases [12]. A study showing no association between LOY and hematological cancers has also been published [13].

An association of LOY has been reported in cancer cells from urothelial bladder cancer [14], pancreatic cancer [15], esophageal carcinoma [16], head and neck carcinoma [17], renal cell carcinoma [18] and in cancer cell lines of hepatocellular carcinoma [19]. These studies have focused on LOY in the tumor itself as a marker for cancer subtype and/or disease progression and possible outcome.

Several studies have investigated mosaic LOY in peripheral blood cells in relationship to presence of disease. An association was found with primary biliary cirrhosis [20], autoimmune thyroiditis [21], but not with male breast cancer [22]. Large cohort SNP array-based studies found an association of mosaic LOY in peripheral blood cells with increased risk of cancer, shorter survival and smoking [23,24].

We here present our results of an association study of LOY in peripheral blood cells in male patients with colorectal and prostate cancers.

## Materials and Methods

### Subjects

We studied 101 male patients with colorectal cancer (mean age 60.5±11.9 yrs), 70 male patients with prostate cancer (mean age 68.8±8.0 yrs) and 93 healthy males from the general population (mean age 65.8±16.6 yrs). Cancer subjects were recruited from the Department of Urology, Medical Faculty, Skopje, Republic of Macedonia and were referred to the Pharmacogenetic Laboratory, Faculty of Pharmacy, while DNA samples from healthy controls were obtained from the DNA bank at the RCGEB "Georgi D. Efremov". Some clinicopathological characteristics of the tumors of the cancer patients are given in Table 1. The study was approved by the Ethics Committee of the Macedonian Academy of Sciences and Arts (Approval No. 2010/1) and the Ethics Committee of the Faculty of Pharmacy, University Ss Cyril and Methodius (Approval No. 03–95), Skopje, Republic of Macedonia. All subjects have given written informed consent for participation in the study according to Declaration of Helsinki.

**Table 1:** Clinicopathological characteristics in colorectal and prostate cancer subjects analyzed in this study.

**Colorectal cancer (n = 101)**	
***Localisation***	n (%)
Proximal	23 (22.8%)
Distal	25 (24.7%)
Rectal	44 (43.2%)
No data	9 (8.9%)
***Stage***	
I	7 (6.9%)
II	29 (28.7%)
III	34 (33.7%)
IV	7 (6.9%)
No data	24 (23.7%)
**Prostate cancer (n = 70)**	
***Gleason sum score***	
5–6	19 (27.1%)
7	26 (37.1%)
8–10	23 (32.9%)
No data	2 (2.9%)

### DNA isolation

DNA was isolated from whole blood. After lysis, the leukocytes were pelletted and digested with proteinase K and DNA was extracted following the standard phenol-chloroform protocol.

### Determination of mosaic LOY in blood

Presence of LOY was determined with multiplex quantitative fluorescence polymerase chain reaction (QF–PCR) originally developed for detection of microdeletions in AZF regions of Y chromosome and sex chromosome aneuploidies in patients with male infertility [25,26]. Relative amount of Y chromosome was assessed through the chromosomeY/chromosomeX (Y/X) ratio of fluorescent signal of co-amplified short sequences from Y-X homologous amelogenin genes (*AMELY* and *AMELX*). *AMELX* contains a 6 base pair (bp) deletion in intron 1 that is missing in *AMELY*, so that co-amplified fragments are sized 112 bp from chromosome Y and 106 bp from chromosome X and are easily separated and quantified by capillary electrophoresis (~1:1 ratio of 112/106 bp peaks in normal male DNA samples and presence of only 106 bp peak in female DNA samples) [27]. False positive results in case of loss or gain of chromosome X were controlled by amplification of 152 bp fragment of the *TAF9B* gene on chromosome X with primers which also co-amplify 148 bp homologous sequence on chromosome 3. The ratio of the peak areas of the two co-amplified fragments (148/152 bp; chromosome 3/chromosome X) is ~2:1 in normal male DNA samples and ~1:1 in normal female DNA samples.

**Fig 1:**
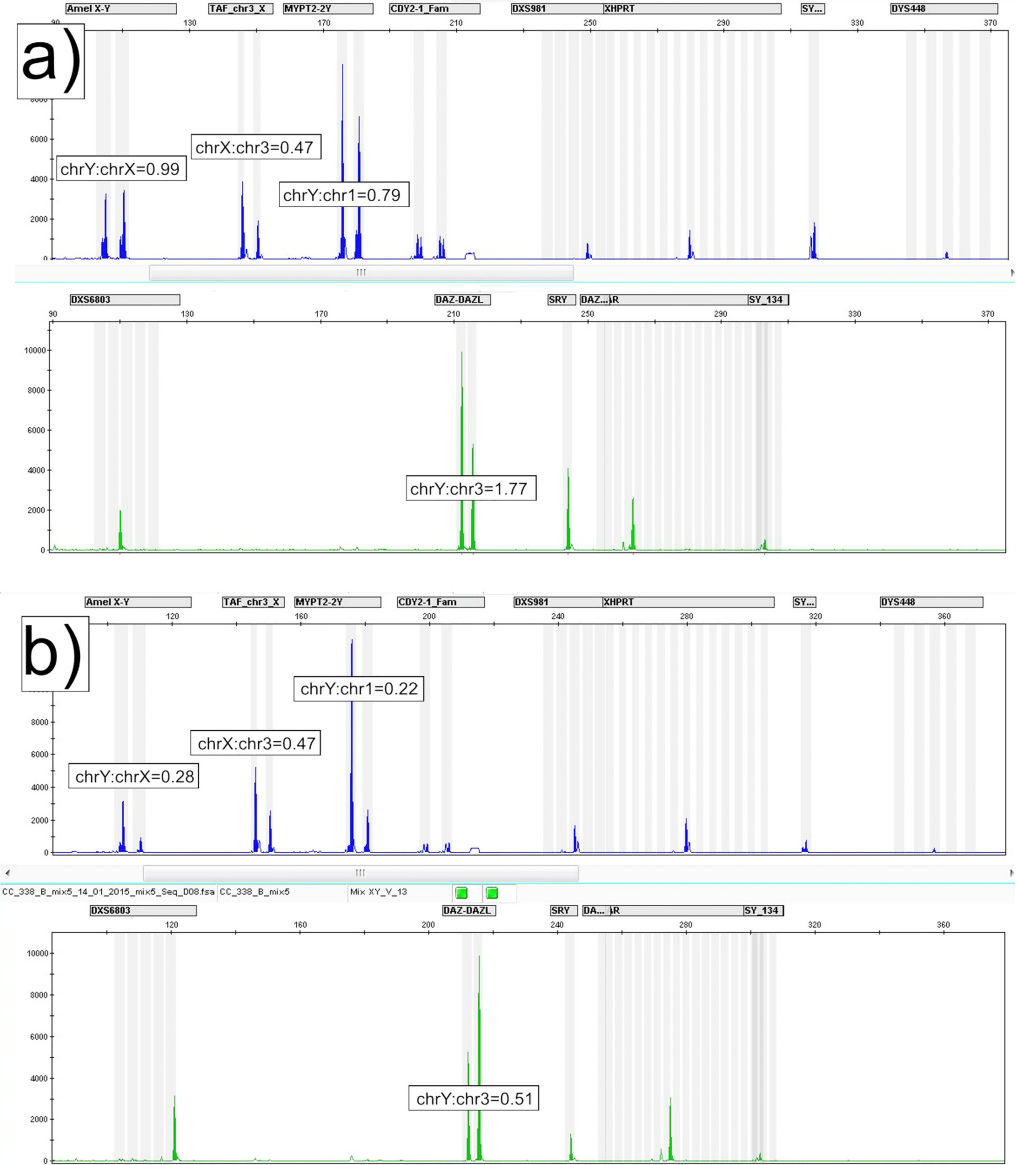
Electrophoregrams of co-amplified homologous sequences. Electrophoregrams from subjects with a) normal amelogenin Y/X ratio (approximately equal peak areas of X chromosome and Y chromosome sequences) and b) patient with colorectal cancer and significantly increased proportion of cells with LOY (amelogenin Y/X ratio of 0.28).

Deletions or duplications in *AMELY* region were excluded by two other primer sets. The first one amplifies homologous sequences of *DAZL* gene on chromosome 3 (two copies of 217 bp fragment) and *DAZ* genes on chromosome Y (four copies of 214 bp fragment). The other primer set amplifies the *MYPT2* gene on chromosome 1 (175 bp fragment) and two homologous sequences (180 bp fragments) from chromosome Y. Primer sequences and PCR conditions for multiplex QF-PCR were as described elsewhere [26]. Co-amplified fluorescently-labeled PCR products were run on Applied Biosystems 3500 Genetic Analyzer with LIZ500 as internal standard and analyzed with GeneMapper 4.1 software (Life Technologies, Carlsbad, CA, USA). Representative electrophoregrams from two subjects, one with with amelogenin Y/X ratio of ~1 and one with amelogenin Y/X ratio of 0.28 are shown in Fig 1.

In order to validate the QF-PCR methodology we have used two different DNA samples: one normal male sample (46, XY), and one sample with Turner syndrome (45,X0) (to mimic the cells with LOY). Both samples were mixed in different proportions to represent samples with 2.5%, 5%, 10%, 15%, 20%, 30%, 50%, 70% and 90% LOY. Prior to the analysis both samples were adjusted to the same concentration. Nucleic acid concentration and sample purity were determined by NanoVue™ Plus Spectrophotometer (GE Healthcare, Little Chalfont, United Kingdom), while precise double stranded DNA concentration was measured with Qubit^®^ dsDNA HS assay kit on a Qubit 2.0 Fluorometer (Thermo Fisher Scientific, Waltham, Massachusetts, USA). With both measurements DNA concentrations and purity for both samples were identical.

The reproducibility of the Y/X ratio determination was measured by repeating the QF-PCR amplification and analysis in 11 subjects with amelogenin Y/X ratio below 0.80. Additionally, the coefficient of variation of the amelogenin Y/X ratio was calculated from the repeated QF-PCR analyses performed in 14 subjects with Y/X ratio between 0.8–1.0 and 12 subjects between 1.0–1.2. To eliminate the possible bias of different PCR set ups, PCR reactions for one third of healthy controls and two thirds of cancer patients were carried out simultaneously in each of the PCR runs.

### Statistical analysis

Differences between means of two groups were analyzed with Student’s t test after assessing for the equality of variances with Levene`s test. Correlation between two variables was analyzed using Pearson correlation coefficient (r). Limit of detection was calculated based on the standard deviation of the response and the slope of a regression line [28]. Differences between means of repeated measurements were analyzed with Paired samples t test. Multivariate logistic regression was used to model the association of LOY and age variables with cancer presence. Level of significance was considered when p value was smaller than 0.05. Coefficient of variation from duplicate measurements was computed with MedCalc for Windows, version 12.5 (MedCalc Software, Ostend, Belgium) according to their described methodology. Data analyses for all other statistical tests were performed using Statistical Package for Social Sciences, Version 19 (SPSS, Chicago, IL, USA).

## Results

### Validation of the amelogenin Y/X peak ratio as a method for detecting mosaic LOY in blood cells and testing for reproducibility

Normal 46,XY DNA sample and nine mixed DNA samples with different proportions of 46,XY and 45,X0 DNA samples were amplified in triplicate and corresponding amelogenin Y/X ratio was obtained for each proportion. Correlation analysis based on averaged values of triplicate measurements showed strong negative correlation (r = -0.999, p = 8.03x10^-13^) between percentage of 45,X0 sample and amelogenin Y/X ratio.

**Fig 2:**
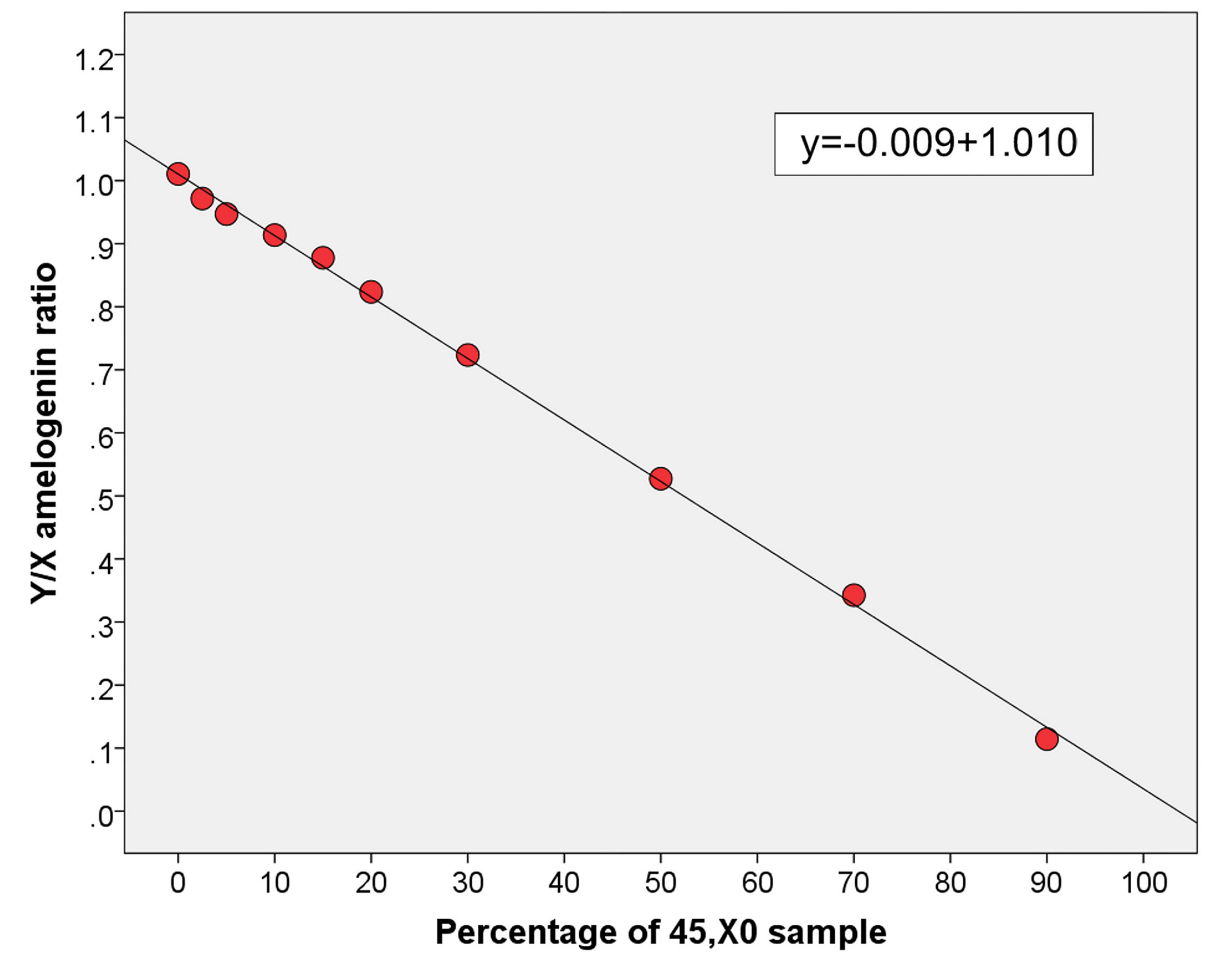
Scatter plot of amelogenin Y/X ratio obtained from different proportions of 45,X0 and 46,XY DNA samples. Dots represent averaged amelogenin Y/X ratio values corresponding to 0%, 2.5%, 5%, 10%, 15%, 20%, 30%, 50%, 70% and 90% of the 45,X0 in the mixed DNA samples. Regression line was estimated using percentage of 45,X0 sample as independent and amelogenin Y/X ratio as dependent variable. Slope (s) and standard deviation (σ) of the regression line were used to calculate the limit of detection using the formula LOD = 3.3σ/s [28].

The limit of detection (LOD) of LOY was calculated at 4.6%, using standard deviation and slope of the regression line of the averaged values of triplicate measurements (Fig 2).

The reproducibility of the amelogenin Y/X ratios was assessed in 37 subjects. The low coefficient of variation of 4.62% and no significant difference in the means between two measurements (p = 0.1097, Paired T test) confirm that QF-PCR is a reliable method for estimation of the amelogenin Y/X ratios.

### Mosaic LOY in blood cells is significantly associated with cancer presence

**Fig 3:**
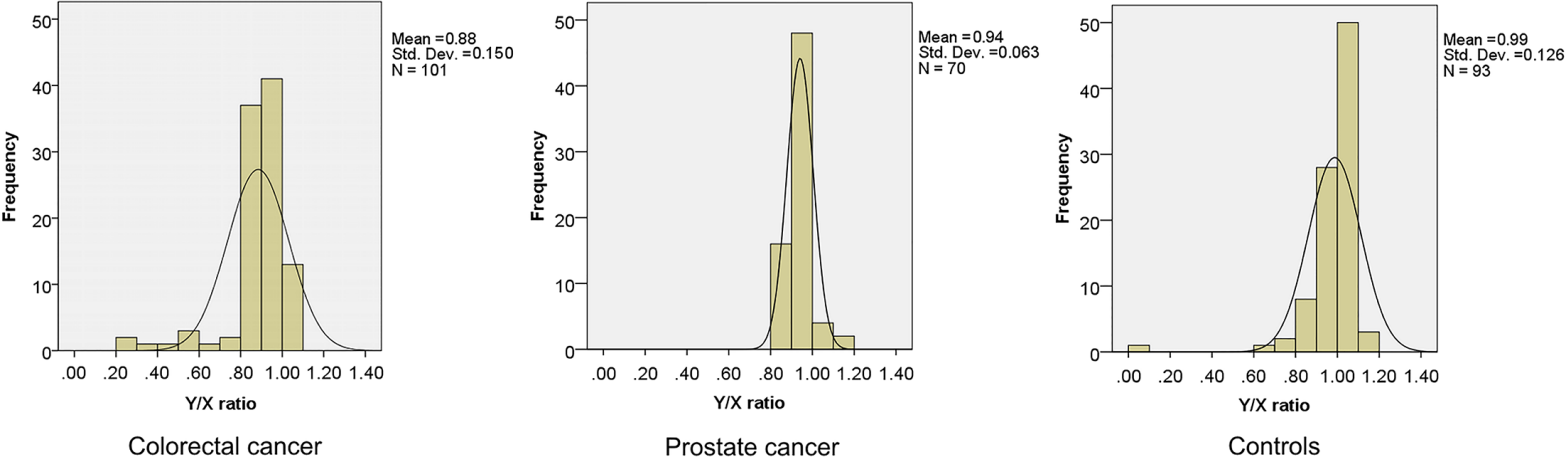
Histograms of distribution of Y/X ratios in colorectal and prostate cancer patients and controls. Mean values and standard deviations are also shown.

We have analyzed the Y/X ratio using QF-PCR in 264 DNA samples isolated from leukocytes from peripheral blood in patients with colorectal cancer (n = 101), prostate cancer (n = 70) and healthy controls (n = 93), matched for age. Distribution of the Y/X ratios is presented as histograms in Fig 3. The mean Y/X ratio was significantly lower in cancer patients (0.907±0.12; p = 1.17x10^-9^) than the controls (1.015±0.15). Statistical significance was retained also when mean Y/X ratios of colorectal patients (0.884±0.15; p = 3.76x10^-9^) and prostate cancer patients (0.941±0.06; p = 1.15x10^-4^) were analyzed separately.

Using multivariable logistic regression we have modeled percentage of LOY (inferred from amelogenin Y/X ratio) and age to assess their strength to predict cancer presence. Both predictor variables were used as continuous variables while outcome was cancer presence and no cancer as reference (Table 2). LOY percentage predicts cancer presence with a significant strenght (p = 2.043x10^-9^), while age showed only borderline significance (p = 0,024). The two predictor variables in the model suggest that increase in age when adjusting for LOY percentage does not increase risk for cancer presence in our cohort of cancer patients.

**Table 2:** Multivariable logistic regression analysis for cancer presence adjusted for age and LOY (measured with amelogenin Y/X ratio).

	Regression coeficient (B)	Significance	Odds ratio	95% Confidence Interval	
				Lower	Upper
LOY percentage	0.107	2.043E-09	1.113	1.085	1.153
Age	-0.026	0.024	0.975	0.953	0.997
Constant	1.877	0.011	6.537		

## Discussion

A number of research groups are investigating non-invasive cancer biomarkers to improve the early diagnosis and management of cancer patients. Recently, LOY was suggested as a possible biomarker for different cancers in males. Data from large cohorts associated LOY in peripheral blood with risks of all-cause mortality and non-hematological cancer mortality [23]. Confirmatory case control studies associating mosaic LOY in peripheral blood cells with increased risk of cancer are needed.

To contribute to the clarification of the role of the LOY in peripheral blood as a cancer biomarker in males, we have performed case control study in male patients with two different types of cancer.

The methodology we used was based on indirect determination of the LOY presence by QF–PCR, measuring the ratio of peak areas of fluorescently labeled PCR fragments of fixed lengths representing homologous sequences on Y chromosome and some other chromosome. This methodology of measuring the ratio of peak areas is widely used in rapid prenatal diagnosis of common chromosome aneuploidies [29]. It is also used for detection of sex chromosomes aneuploidies as well as for deletions and rearrangements in AZF regions on Y chromosome [25]. In contrast to the cytogenetic analysis of cultured blood cells (only T lymphocytes) which is time consuming and prone to technical artifacts such as chromosome loss during slide preparation [3], QF-PCR methodology is performed on a DNA isolated from leukocytes from whole blood, and it is inexpensive and more robust. We were unable to compare results of amelogenin Y/X ratios using direct counting of cells with cytogenetic analysis, but we have validated our methodology by simulating LOY with the use of 45,X0 DNA sample. The high correlation between 45,X0 percentage and amelogenin Y/X ratio confirms that QF-PCR is a reliable method for estimation of the Y/X ratios. Although there are reports for *AMELY* allelic dropout as a result of the deletion in this region of Y chromosome, the increased frequency is mainly reported in Asian populations [30,31,32]. We have not detected loss of *AMELY* among males included in this study as well as more than 4000 male samples analyzed in our laboratory for the presence of the most common chromosomal aneuploidies, paternity and male infertility (unpublished data).

In our study, we included male patients with prostate and colorectal cancer. Although the amelogenin Y/X ratio does not give a precise value of the percentage of cells with LOY, the mean ratio values obtained within same experimental conditions represent an approximate reflection for extent of LOY for the studied groups. The mean Y/X ratios were significantly lower in colorectal patients than prostate cancer patients. The higher percentage of patients with LOY in our cohort of cancer patients is consistent with the recent findings showing significantly increased frequency of sex chromosomes monosomies in patients with lung and bladder cancer in comparison to healthy controls [33].

Because LOY is reported to be associated with age, we have matched our cancer patients and control non-cancer subjects with age and subsequently modeled age and LOY percentage for cancer presence predictions. The results showed that increased number of subjects with LOY in our cohort of cancer patients probably is not influenced by their age.

The exact mechanism by which cells are losing Y chromosome is not known. One proposed mechanism is the influence of telomere shortening during aging which leads to grater instability and degradation of Y chromosome which has shorter telomeres than autosomes [34]. Another proposed mechanism is related to the property of the Y chromosome to replicate late in S-phase [21], thus shortening of the cell cycle (and losing Y chromosome) could give a proliferative advantage to a disease affected organism. Different pathways might be activated, leading to different proliferation rates and number of hematopoietic cells with LOY in different cancers in response to signals from cancer cells. Variation in the genome between different cancer populations could additionally modulate the response of hematopoietic cells to cancer cell signals. Additional evidence for this proposed mechanism of signal induced LOY is related to the fact that smoking has a transient and dose-dependent mutagenic effect on LOY-status [24]. Also, this mechanism is in agreement with the proposed influence of altered tissue microenvironmental signaling on somatic evolution in an age-dependent manner [35]. Recently, clonal hematopoiesis with somatic mutations was shown not only to be increasingly common in elderly people but also to increase the risk of hematological malignancies and death [36,37], with the possibility that all-cause mortality is due to an increased risk of cardiovascular disease [36]. These studies analyzed only single nucleotide variants and small indels and do not report large aberration like Y chromosome loss. Also, large chromosomal abnormalities (>2Mb) of autosomes in DNA from blood were found to be more frequent among individuals with solid tumors than in cancer-free individuals [38]. It would be interesting to see if occurrence of LOY, somatic mutations and/or certain types of chromosomal abnormalities are linked and present together in same cells or cell types. If health impairment promotes simultaneous occurrence of different types of genomic mutations, the easily detectable mosaic LOY could become a valuable biomarker.

In conclusion, results from our case-control study showed strong link between cancer presence and mosaic LOY in peripheral blood of men affected with colon and prostate cancer being the two most common solid tumors in men. They also support recent findings of association of LOY in blood with carcinogenesis in males.
